# Are Countries of the Eastern Mediterranean Region on Track towards Meeting the World Health Assembly Target for Anemia? A Review of Evidence

**DOI:** 10.3390/ijerph18052449

**Published:** 2021-03-02

**Authors:** Ayoub Al-Jawaldeh, Mandy Taktouk, Radhouene Doggui, Zahra Abdollahi, Baseer Achakzai, Hassan Aguenaou, Moussa Al-Halaika, Salima Almamary, Rawhieh Barham, Ferima Coulibaly-Zerbo, Laila El Ammari, Jalila Elati, Noureen Aleem Nishtar, Nasrin Omidvar, Mohammad Qasem Shams, Abdul Baseer Qureshi, Lara Nasreddine

**Affiliations:** 1 Regional Office for the Eastern Mediterranean (EMRO), World Health Organization (WHO), Cairo 7608, Egypt; aljawaldeha@who.int; 2 Nutrition and Food Sciences Department, Faculty of Agriculture and Food Sciences, American University of Beirut, Beirut 11-0236, Lebanon; mt86@aub.edu.lb; 3 Family Medicine Department, Université de Sherbrooke, Sherbrooke, QC J1K 2R1, Canada; radhouene.doggui@gmail.com; 4 Medical Research Department, Centre de Formation Médicale du Nouveau-Brunswick, Moncton, NB E1A 7R1, Canada; 5 General of Nutrition Department, Ministry of Health and Medical Education, Tehran 15655-415, Iran; abdollahi_z@yahoo.com; 6 Nutrition/Regulations, Ministry of Health, Islamabad 56000, Pakistan; achakzaib@gmail.com; 7 Joint Research Unit in Nutrition and Food, RDC-Nutrition AFRA/IAEA, Ibn Tofaïl University-CNESTEN, Rabat-Kénitra, Rabat-Kénitra 242, Morocco; AGUENAOU.Hassan@uit.ac.ma; 8 Nutrition Department, Ministry of Health, Ramallah 4284, Palestine; mosahalaika@gmail.com; 9 Nutrition Department, Ministry of Health, Muscat 393, Oman; Dr.salima.almamary@gmail.com; 10 Nutrition Department, Ministry of Health, Amman 11118, Jordan; majeda_barham@hotmail.com; 11 Nutrition Team Lead WCO, World Health Organization (WHO), Sana’a 543, Yemen; zerbof@who.int; 12 Nutrition Department, Ministry of Health, Rabat 335, Morocco; elammarilaila0@gmail.com; 13 SURVEN (Nutrition Surveillance and Epidemiology in Tunisia) Research Laboratory, INNTA (National Institute of Nutrition and Food Technology), Tunis 1007, Tunisia; jalila.elati@rns.tn; 14 Nutrition Department, World Health Organization (WHO), Islamabad 1013, Pakistan; nishtarn@who.int; 15 Community Nutrition Department, National Nutrition and Food Technology Research Institute, Faculty of Nutrition Sciences and Food Technology, Shahid Beheshti University of Medical Sciences, Tehran 19395-4741, Iran; omidvar.nasrin@gmail.com; 16 Nutrition Department, World Health Organization (WHO), Kabul, Jalalabad Road Pul-e-Charkhi Kabul, Afghanistan; shamsm@who.int; 17 Country Office, World Health Organization (WHO), Khartoum 2234, Sudan; qureshiab@who.int

**Keywords:** iron deficiency anemia, underfive children, women, pregnant women, women of reproductive age, nutrition-specific interventions, non-nutritional causes, anemia targets

## Abstract

Anemia is a multifactorial condition, with a complex etiology that involves nutritional and non-nutritional factors. The misconception that iron deficiency is equivalent to anemia may mask the need to address other potential causative factors. This review paper aims to (1) assess the burden of anemia vs. iron deficiency anemia (IDA) amongst women of reproductive age (WRA), pregnant women (PW), and children under five years old (underfive children, U5C) in the Eastern Mediterranean region (EMR); (2) evaluate trends in anemia prevalence and whether countries are on track towards meeting the World Health Assembly (WHA) target for 2025; and (3) characterize anemia reduction efforts and provide a road map for future programs. A search of pertinent literature and databases was conducted. Anemia prevalence in the EMR ranged between 22.6% and 63% amongst PW, 27% and 69.6% amongst WRA, and 23.8% and 83.5% amongst U5C. Data showed that the EMR is not on course towards meeting the WHA target. The contribution of IDA to anemia was found to be less than half. Other potential contributors to anemia in the region were identified, including micronutrient deficiencies, parasitic infestations, and poor sanitation. A framework of action was proposed as a roadmap to meet the targets set by the WHA.

## 1. Introduction

Anemia is recognized as a global public health concern, affecting women of reproductive age (WRA), pregnant women (PW), adolescent girls, and young children, particularly in low- and middle-income countries [1,2]. Anemia is defined as the condition of having a low concentration of hemoglobin (Hb) or a low number of red blood cells [2,3]. Clinically, it is classified as mild, moderate, or severe based on Hb concentrations, the cutoffs being dependent on age, sex, and physiological state. The reduction in Hb levels that characterizes anemia limits blood oxygen transport, making it insufficient to meet the body’s physiologic needs [4]. This results in reduced physical and mental capacity, while also increasing the risk for other adverse health impacts [2,3,5]. In children and adolescents, anemia may impair physical growth and cognitive development, reduce physical fitness and school performance, and increase the risk of infections [6,7,8]. In WRA, anemia results in fatigue, dizziness, reduced work capacity, loss of productivity, and increased susceptibility to infections [9,10,11]. During pregnancy, anemia increases the risk of serious health consequences for both the mother and the neonate, including miscarriage, stillbirth, preterm delivery, intrauterine growth retardation, low birth weight, and mortality [12,13]. Non-health consequences of anemia encompass increased healthcare expenditures and reduced income, with their associated impacts on families and communities [2,3]. Thus, reducing anemia across the lifecycle is recognized as a crucial factor in the enhancement of women’s and children’s health, school performance, work productivity, healthier pregnancy, and birth outcomes, while allowing for intergenerational benefits for health, economy, and community development [14,15]. The reduction of anemia is specified as one of the World Health Assembly (WHA) Global Nutrition Targets for 2025 [11], and is included in the Sustainable Development Goals (SDGs) targets and indicators for nutrition [16]. The targets focus particularly on WRA, stipulating a 50% reduction in the prevalence of anemia in this population group by the year 2025 (compared to 2012 as the baseline) [11].

Anemia is a multifactorial condition, with a complex etiology that involves nutritional and non-nutritional factors and mechanisms [2,17]. Amongst the well-documented causes of anemia, iron deficiency is undoubtedly the most common, with the terms anemia, iron deficiency, and iron-deficiency anemia (IDA) often being used interchangeably [2,18]. However, although iron deficiency results in reduced Hb levels and decreased erythrocyte production, there are numerous other causes of anemia that do not involve iron deficiency [2,5,17,19]. Given the complex etiology of anemia, successful and effective anemia reduction efforts should not focus solely on iron, but rather should identify all additional contributing factors in order to develop and implement an evidence-based set of interventions, tailored to the context and to the locally identified determinants [2]. A conceptual model, adapted by Chaparro and Suchdev [17], has categorized the various factors contributing to anemia into the (1) fundamental determinants (political, economy, ecology, climate, and geography); (2) underlying determinants (education, wealth, social norms, and health systems, coupled with the physiological vulnerability of women and children); (3) intermediate determinants (food insecurity, inadequate maternal and child care, limited access to health and nutrition services, inadequate health and nutrition knowledge, and inadequate access to water sanitation and hygiene); and (4) immediate determinants that include inadequate nutrient intake/utilization, chronic exposure to infectious diseases, micronutrient deficiencies, inflammation, and genetic Hb disorders [17,19,20,21]. In a recently published report, the World Health Organization (WHO) has classified the causes of anemia as nutritional vs. non-nutritional causes. The first category includes iron deficiency and other concurrent or separate micronutrient deficiencies, such as vitamin A, riboflavin (B2), cobalamin (B12), pyridoxine (B6), folate (B9), and vitamins C, D and E, as well as mineral elements, including copper and zinc [22]. The non-nutritional causes include parasites, infections, inflammation, and genetic disorders, as well as environmental and socioeconomic contributors to anemia, including water, sanitation, and hygiene (WASH), climate change and environmental pollutants, women’s empowerment and poverty, education, and access to care [22].

The Eastern Mediterranean Region (EMR), which harbors countries with significant disparities in socioeconomic and developmental landscapes, is a region that suffers from a high burden of anemia in women and children. Within the region, many countries have continued to move through the nutrition and epidemiological transition, while others have witnessed significant increases in undernutrition, micronutrients deficiencies, and anemia as a result of persistent conflict and political instability. The Regional Strategy on Nutrition 2010–2019 and Plan of Action [23] aimed to tackle the burden of anemia in the EMR, with a target of 30% reduction in the prevalence of IDA amongst children and WRA. The 2020–2030 Strategy on Nutrition for the EMR adopted the WHA target of reducing anemia by 50% in WRA [24]. Studies reporting on the regional progress in anemia reduction are lacking. In addition, little is known on anemia reduction strategies and interventions being implemented in EMR countries and whether these programs focus solely on IDA or aim to holistically tackle the multifactorial nature of anemia. In this context, the objectives of this review are to (1) assess the burden of anemia vs. IDA amongst WRA, PW, and underfive children (U5C) in the EMR; (2) evaluate the trend in anemia prevalence and whether countries are on track towards meeting the WHA target for 2025; and (3) characterize anemia reduction efforts and provide a road map for future programs.

## 2. Approach

This review is based on a comprehensive search of the literature, including national/regional studies and review articles published between 1995 and September 2020, and the retrieval of data from the Global Health Observatory (GHO), the Global Database on the Implementation of Nutrition Action (GINA) [25], the WHO EMRO (Regional Office for the Eastern Mediterranean) website [26], and governmental websites.

Data on the prevalence and temporal trends of anemia in PW, WRA, and U5C in countries of the EMR (*n* = 22 countries) were retrieved mainly from the GHO of the WHO [27,28], starting with the year 2010 and including the most recent available estimates, which pertained to the year 2016. The researchers accessed the GHO database between November 2020 and January 2021. In some cases where data were incomplete in the GHO database or where more recent national estimates were available, data was supplemented from the WHO regional frameworks [29,30] or national reports published by country-specific governmental entities, when available. Anemia was defined based on Hb levels below 110 g/L for U5 children and PW and below 120 g/L for WRA and adolescent girls (adjusted for altitude and smoking).

The interpretation of the severity of anemia as a public health problem was based on population prevalence, as follows [4]: (a) not a public health problem: ≤4.9%; (b) mild: 5.0–19.9%; (c) moderate: 20.0–39.9%; or (d) severe: ≥40.0% of the population affected by the problem. To assess whether countries are on track towards meeting the WHA global target, the percentage change in the prevalence of anemia amongst WRA was calculated when data were available at different points in time. Considering the WHA target of 50% reduction in anemia prevalence from 2012 to 2025 and a linear expected change per year to reach this goal, a relative decrease of at least 15.4% would be expected between 2012 and 2016, or at least 19.2% between 2012 and 2017. A relative decrease of 0% to 15.4% from baseline would be considered as headed in the right direction, whereas countries that experience an increase in the prevalence would be considered off track.

The percentage change in the prevalence of anemia was calculated based on the following formula:

$$\frac{C=P\;\mathrm{recent}-P\;\mathrm{baseline}*100}{P\;\mathrm{baseline}}$$

C = percentage change
P baseline: prevalence estimated based on the baseline survey, i.e., 2012
P recent: prevalence estimated based on the most recent survey

Data on IDA and other micronutrient deficiencies that may be associated with anemia (zinc, copper, folate, and vitamins A, B2, B6, B12, C, D, and E) were gathered where available from published individual studies and review articles. Electronic databases (MedLine, PubMed, Scopus, and Google Scholar) were searched between 1 November 2020 and 30 November 2020 using a pre-determined search strategy relying on combinations of key terms. For the assessment of the prevalence of IDA in Member States, the key terms used in the search included EMR countries and/or each country alone, AND “iron deficiency anemia” OR “anemia”, AND “prevalence” OR “trend”, AND “women” OR “pregnant” OR “underfive children” OR “preschool children” OR “adolescent”. A similar search strategy was adopted for reviewing the other micronutrient deficiencies that may be associated with anemia. The reference lists of the specific studies were also reviewed to identify additional data sources. Studies were included if they were performed after 1995. In addition, a search of governmental websites, such as those of the ministries of health or national public health institutes, was performed in order to identify additional data on IDA, as well as micronutrient deficiencies. IDA was defined based on Hb and serum ferritin levels (SF), as follows: U5C: Hb < 110 g/L and SF < 12 µg/L; WRA and adolescent girls: Hb < 120 g/L and SF < 15 µg/L; and PW: Hb < 110 g/L and SF < 15 µg/L [4,31].

For the national anemia reduction policies, programs, or interventions in countries of the EMR, information was retrieved from published individual studies and review articles using a pre-determined search strategy, as well as from the WHO GINA [25] and the WHO EMRO website [26]. In addition, a search of governmental websites was performed for additional data.

## 3. Prevalence of Anemia

### Prevalence of Anemia in WRA, PW, and U5C in Countries of the EMR

Table 1 presents country-specific data on the prevalence of anemia amongst PW, WRA, and U5C based on the most recent data available in the GHO [27,28]. In some cases where more recent national estimates were available, data was supplemented from the WHO regional frameworks [29,30] or national reports published by country-specific governmental entities. The prevalence of anemia amongst PW (Hb < 110 g/L) ranged between 22.6% and 63% in countries of the region. Anemia prevalence was found to exceed 40% in Yemen (63%), Pakistan (51.3%), Somalia (46.8%), Saudi Arabia (45.5%), Bahrain (42.8%), and Morocco (40.4%) [27], highlighting a severe public health problem.

In WRA, the prevalence of anemia (Hb < 120 g/L) ranged from 27% to 69.6% (Table 1). Based on the prevalence estimates listed in Table 1, anemia is identified as a severe public health problem in six countries of the region, including Yemen (69.6%), Bahrain (45.9%), Somalia (44.4%), Saudi Arabia (43.7%), Pakistan (42.7%), and Afghanistan (42%) [30]. Data on anemia amongst adolescent girls are scarce, but available data highlight the need to focus on this age group in future public health interventions. In Pakistan, the National Nutrition Survey (NNS) (2018) showed that more than half (56.6%) of adolescent girls (10–19 years) were anemic [32], while in Afghanistan, anemia was identified in around a third of adolescent girls (30.9%) [33]. The National Micronutrient Survey in Iran showed that 12.1% of girls aged 14–20 years were anemic at the national level, while in deprived Southern provinces, anemia prevalence increased to 26% [34]. In Palestine (Gaza Strip) and Oman, the prevalence of anemia amongst adolescent girls (15–19 years old) was reported at 35.8% [35] and 28.8% [36], respectively.

In U5C, the prevalence of anemia (Hb < 110 g/L) ranged between 23.8% and 83.5% (Table 1). Estimates exceeding 40% were reported in Yemen (83.5%), Somalia (55.8%), Pakistan (53.7%), Sudan (48.1%), Morocco (47.5%), Afghanistan (46.4%), and Djibouti (42%) [28].

## 4. Are Countries Making Progress in Reducing the Prevalence of Anemia over Time?

In 2012, the WHA set a global target of reducing anemia amongst WRA by 50% in 2025 [11]. Data from the WHO GHO [40] show that, instead of decreasing, the regional prevalence of anemia in the EMR has increased from 37.8% in 2012 to 39.8% in 2016 amongst WRA and from 40.2% to 40.9% amongst PW during the same time period [27].

**Figure 1:**
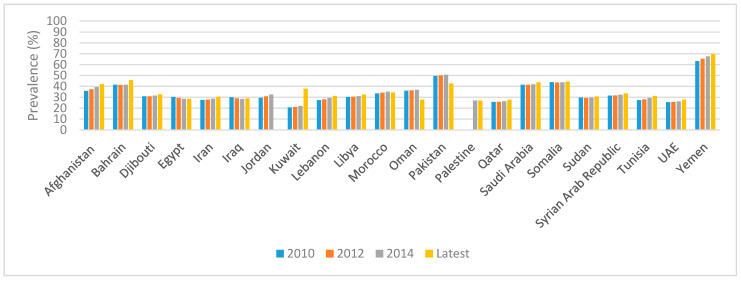
Trends in the prevalence of anemia amongst WRA (15–49 years) in countries of the EMR. References: 2010, 2012, and 2014 data were based on the WHO GHO Database [27]. The latest data were based on the WHO GHO Data Repository–Prevalence of anemia in women (2016) [27] and on the WHO EMRO Monitoring health and health system performance in the Eastern Mediterranean Region, Core indicators and indicators on the health-related Sustainable Development Goals 2019 [30], with the following exceptions: the data for Morocco were based on the recent Moroccan NNS 2019–2020 [37]. The data for Oman were based on the recent Oman NNS in 2017 [36]. The data pertinent to Pakistan were based on the recent NNS 2018 [32]. Anemia was defined as an Hb concentration < 120 g/L, adjusted for altitude and smoking. Abbreviations: EMR: Eastern Mediterranean Region; EMRO: Regional Office for the Eastern Mediterranean; GHO: Global Health Observatory; Hb: hemoglobin; NNS: National Nutrition Survey; UAE: United Arab Emirates; WHO: World Health Organization; WRA: women of reproductive age.

Figure 1 displays the temporal trend in anemia prevalence amongst WRA in various countries of the region. Available data show that, in most countries of the region, the prevalence of anemia has either increased or remained stagnant over time, and, therefore, the majority of countries are off track in comparison with the WHA target (Figure 1). Amongst the countries in which there has been some progress toward reducing anemia, Oman, Pakistan, and the Syrian Arab Republic were found to be on track towards meeting the WHA target (−23.4% between 2017 and 2012; −15% between 2018 and 2012; −22.7% between 2016 and 2012, respectively), and one country (Egypt) can be considered in the right direction (−2.7% between 2016 and 2012).

**Figure 2:**
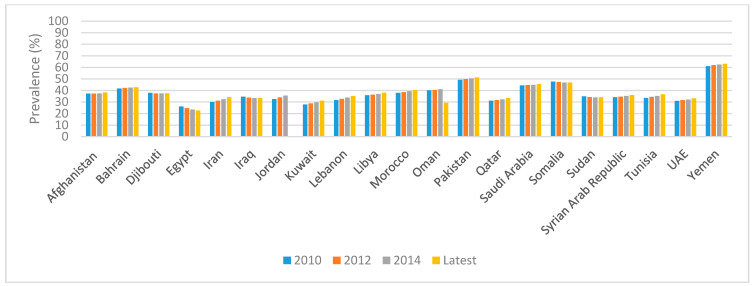
Trends in the prevalence of anemia amongst PW in countries of the EMR. References: The 2010, 2012, and 2014 data were based on the WHO GHO Database [27]. The latest data were also based on the 2016 WHO GHO Database [27], with the exception of the data pertinent to Oman, which were based on the recent Oman NNS 2017 [36]. Anemia was defined as an Hb concentration <110 g/L, adjusted for altitude and smoking. Abbreviations: EMR: Eastern Mediterranean Region; GHO: Global Health Observatory; Hb: hemoglobin; NNS: National Nutrition Survey; PW: pregnant women; UAE: United Arab Emirates; WHO: World Health Organization.

**Figure 3:**
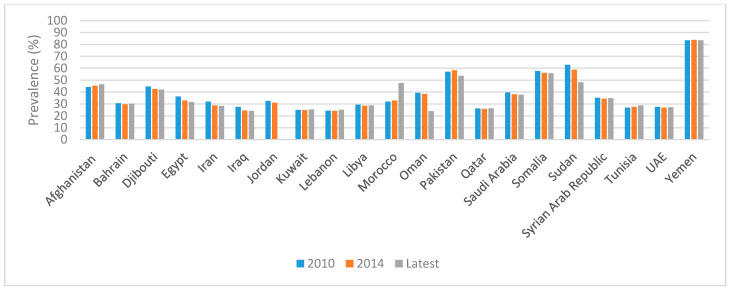
Trends in the prevalence of anemia amongst U5C in countries of the EMR. References: The 2010 and 2014 data were based on the WHO GHO Database [28]. The latest data were also based on the 2016 WHO GHO Database [28], with the exception of those for Morocco, Oman, Pakistan, and Sudan; they were based on the recent Moroccan NNS 2019–2020 [37], Oman NNS 2017 [36], Pakistan NNS 2018 [32], and Sudan Simple Spatial Survey Method (S3M II) [39], respectively. Anemia was defined as an Hb concentration < 110 g/L, adjusted for altitude. Abbreviations: EMR: Eastern Mediterranean Region; GHO: Global Health Observatory; Hb: hemoglobin; NNS: National Nutrition Survey; U5C: underfive children; UAE: United Arab Emirates; WHO: World Health Organization.

Data specific to PW show that the prevalence of anemia has remained stagnant over time in all countries of the region, except for Oman and Egypt, which have experienced a relative decreasing trend (Figure 2). As for U5C, the prevalence of anemia in this age group has been stagnant over time in most countries of the region, with only Egypt, Iran, Iraq, Oman, and Sudan making some progress (Figure 3).

Available data, therefore, show that most countries of the region are not on track towards meeting the 2025 WHA target for WRA, and the majority are not making progress in reducing anemia in other population groups, including U5C.

## 5. Why Are EMR Countries Not Making Progress in Anemia Reduction? The Need to Understand the Local Context

In order to gain a better understanding as to why adequate progress is not being made in countries of the region, it is important to understand the extent to which different factors, nutritional and non-nutritional, may be contributing to the burden of anemia in the EMR, and identify the main anemia reduction strategies being implemented by countries of the region.

### 5.1. What Is the Contribution of Iron Deficiency to Anemia in Countries of the EMR?

**Figure 4:**
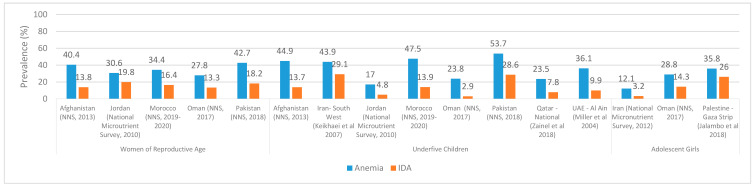
Prevalence of IDA vs. anemia amongst WRA, U5C, and adolescent girls based on studies that have reported on both. For Afghanistan, Iran, Jordan, Qatar, and the UAE, the values for WRA and U5C may differ from those reported in Table 1, since the values in the figure are based on studies that have reported on both IDA and anemia. Abbreviations: EMR: Eastern Mediterranean Region; IDA: iron deficiency anemia; NNS: National Nutrition Survey; U5C: underfive children; UAE: United Arab Emirates; WRA: women of reproductive age.

The relative contribution of IDA to anemia in the EMR was assessed based on national surveys or studies that have reported on both indicators on the same population. Overall, data on IDA were not systematically available for EMR countries. Given the high cost and complexity of blood collection, treatment, transportation, storage, and analyses, few countries have collected data on the population’s SF levels, which are used to assess the proportion of anemia that is due to iron deficiency [2,41]. Available evidence suggests that the prevalence of IDA is considerably lower than that of anemia (Figure 4). For instance, in Afghanistan, the NNS estimated the prevalence of IDA at 13.8% in WRA, while that of anemia was three times higher (40.4%) [33]. Similar observations were reported from Jordan, Morocco, Oman, and Pakistan [32,36,37,42] (Figure 4). National studies conducted amongst WRA in Iraq, Jordan, and Lebanon estimated the prevalence of IDA at 4.9%, 19.8%, and 13.4%, respectively [42,43,44]. Amongst PW, available data on IDA is very limited. In Kuwait, it was reported that IDA represented approximately 60% of overall anemia cases in PW, the latter being estimated at 41.3% [45]. A study conducted in rural districts of Egypt reported a prevalence of 51.3% of IDA amongst PW [46]. In Iran, the prevalence of anemia and IDA amongst PW was estimated at 14% and 0.6 %, respectively [34].

In Figure 4, data on IDA amongst adolescent girls are presented, despite their scarcity. The National Micronutrient Survey in Iran reported a low prevalence of IDA amongst adolescent girls, estimated at 3.2%, which is four times lower than the prevalence of anemia in this age group (12.1%) [34]. In Oman, the prevalence of IDA in adolescent girls (14.3%) is approximately half the overall prevalence of anemia in this population group (28.8%) [36]. The prevalence of IDA (Hb < 110 g/L and SF < 12 ng/mL) has also been rarely reported amongst U5C in the EMR. In Afghanistan, IDA was identified in 13.7% of U5C, while the prevalence of anemia was more than three times higher (44.9%) [33]. Similarly, the prevalence of IDA amongst U5C in Jordan, Morocco, Pakistan, Qatar, and the United Arab Emirates (UAE) was two to three times lower than that of anemia [32,37,42,47], while it was ten times lower in Oman. Studies conducted amongst preschool children estimated the prevalence of IDA at 4.8% in Jordan [42], 6.8% in Iraq [43], and 11.1% in Lebanon [44,48]. In Iran, the prevalence of anemia in 15–23 month old children was estimated at 17.1%, and that of IDA at 4.2% [34], while higher IDA prevalence was reported amongst preschoolers from South West Iran (29.1%) [49,50]. Using lower cutoff criteria (Hb < 110 g/L and SF < 10 ng/mL), 9.9% and 26.4% of preschool children were found to have IDA in the UAE (Al Ain) and Yemen (Hodiedah & Taiz), respectively [51,52].

Based on the available data, the relative proportion of anemia that is due to iron deficiency is likely to be less than half (or even less than a third in some countries) (Figure 4).

A comparison of the public health burden of anemia vs. IDA is shown in Table 2 for countries where estimates are available for both. This comparison shows that the public health severity of IDA is lower in all countries and in both age groups (WRA and U5C) compared to anemia in general.

### 5.2. What Do We Know about Other Micronutrient Deficiencies in the EMR?

The main nutritional cause of anemia is iron deficiency, but the deficiency of other micronutrients, including vitamins A, B2, B6, B12, folate, C, D, and E, as well as copper and zinc [2,17,19], may also be a contributing factor. As discussed earlier, the proportion of anemia that is linked to iron deficiency was less than half in most countries of the region, highlighting the need to tackle other causes of anemia. Data on the prevalence of other micronutrient deficiencies that may be associated with anemia etiology are scarce in countries of the region [53,54]. A small number of countries have assessed the prevalence of folate deficiency amongst adolescents and WRA, and there are no data for preschool children and PW. Studies found that 7.4% of adolescents in Afghanistan [33] and 69% of schoolgirls in Sudan [55] were folate deficient. It was also reported that 11.6% of WRA in Oman [36], 11.7% in Morocco [37], 13.6% in Jordan [42], 19% in Iraq [43], and 25.1% in Lebanon [56] had folate deficiency.

The prevalence of vitamin A deficiency (VAD) amongst U5C has been reported to range from 6.4% to 62.4%, with the highest rates reported from Yemen (62.4%), Pakistan (51.5%), Afghanistan (50.4%), and Iraq (41.7%) [33,34,36,37,42,54,57,58,59,60,61,62,63,64,65]. Low prevalence rates of VAD were reported from Oman (9.5%) [36], Egypt (9.3%) [62], Morocco (8.8%) [37], and Kuwait (6.4% of boys and 9.3% of girls) [54], while 18.3% of U5C in Jordan [42] and a third of U5C in Somalia (33.3%) [64] and Palestine (33% in boys and 30.4% in girls) [65] were found to have VAD. Iran reported a VAD prevalence of 18.3% in children under the age of two years [34], but available data suggest that there has been a rise in the prevalence compared to 2000 [63]. The prevalence of VAD amongst PW and WRA was assessed in very few Member States. The highest prevalence rate amongst PW was recorded in Pakistan (46%) [66], with lower estimates reported from the UAE (3%) [67] and Iran (14.1%) [34,63]. Amongst WRA, the burden of VAD varied between countries, with 0.2% in Oman [36], 1.7% in Lebanon [68], 4.8% in Jordan [42], 11.3% in Afghanistan [33], and 27.1% in Pakistan [32].

Few countries have assessed zinc deficiency amongst children and women. National studies conducted in Afghanistan, Iran, and Pakistan found that 15.1%, 28%, and 18.6% of U5C were zinc deficient [32,33,34], respectively, while higher estimates were reported from Palestine (55% in boys and 55.9% in girls) [65]. Amongst PW, 47.6% of PW in Pakistan [66], 38% in Sudan [69], and 28% in Iran [34] were zinc deficient. In WRA, the prevalence of zinc deficiency was estimated at 23.4% in Afghanistan [33] and 22.1% in Pakistan [32]. Data on other micronutrient deficiencies are very limited. For instance, vitamin B12 deficiency was identified in 10.9% of U5C in Palestine [65], while in WRA, the prevalence of vitamin B12 deficiency varied between 8.9% in Oman [36], 11.1% in Jordan [42], and 39.4% in Lebanon [56].

Although the link between vitamin D deficiency (VDD) and anemia remains inconclusive, some studies have suggested an association between VDD and IDA in various population groups [70,71]. Available data suggest that VDD is prevalent in the EMR, with its prevalence ranging between 5% and 81% amongst preschoolers [32,33,36,37,42,65], between 40% and 88.8% amongst PW [66,72,73,74], and between 16.2% and 95.5% amongst WRA [32,33,36,37,42,43,73,75,76,77,78].

Therefore, although data availability is rather limited, existing evidence suggests that micronutrient deficiencies that may be possibly associated with anemia are common, and their burden is not to be neglected.

### 5.3. Interventions to Address Nutritional Causes of Anemia in the EMR

Several interventions and initiatives are being implemented in countries of the EMR to tackle the persistent burden of micronutrient deficiencies and associated anemia. These include fortification, supplementation, and nutrition education programs.

#### 5.3.1. Fortification Programs

Specific WHO recommendations for the control and treatment of anemia include the fortification of wheat flours and staple foods with iron, folic acid, and other micronutrients [11,54]. As shown in Table 3, the majority of countries (18) in the EMR have focused on iron in their flour fortification programs, with iron fortification being mandatory in 13 Member States (Afghanistan, Bahrain, Djibouti, Iran, Iraq, Jordan, Kuwait, Morocco, Oman, Pakistan, Palestine, Saudi Arabia, and Yemen) [54]. Amongst the countries that have made some progress in anemia reduction, Oman and Pakistan implement mandatory iron fortification of wheat flour, while Egypt and the Syrian Arabic Republic have voluntary programs.

Fortification with folate is also mandatory in 13 EMR countries, while fortification with other B vitamins is taking place in eight countries on a mandatory or voluntary basis. In contrast, fortification with vitamin A and zinc is currently rare, limited to three to five countries in each case [54]. Some Member States, including Afghanistan, Egypt, Morocco, Oman, Pakistan, and Yemen, have also expanded their fortification programs to other vehicles, such as oil (Table 3).

#### 5.3.2. Supplementation Programs

Supplementation programs have been described as effective strategies in decreasing the risk of anemia in women and children [79]. Although the use of iron supplements was suggested to increase the risk of infections and hence further contribute to anemia [80], a review of studies conducted amongst non-pregnant WRA showed that modest-dose intermittent iron supplementation, alone or with any other vitamins and minerals, was effective in reducing the risk of anemia by 27% [81]. Another review of daily iron supplementation trials amongst PW reported a 67% reduction in IDA [79,82]. In the EMR, the majority of countries (20) are implementing supplementation programs for PW (iron in all of these countries, folate in 90% of these countries, and multiple micronutrient supplements in 52%) [83]. In addition, eight countries in the region report the provision of supplements (folic acid, iron) to WRA [83].

Data related to the uptake of supplementation programs in the EMR are scarce, and in many instances, available data highlight challenges in program implementation. For instance, the NNS conducted in Afghanistan reported low use of supplements amongst PW, with the most commonly used supplements being iron (23.2%), folic acid (13.8%), and multivitamins (12.4%) [33]. More than half of the women participating in the survey (54.1%) did not remember whether they received any supplements during their last pregnancy [33]. In contrast, a much higher supplement use was reported amongst PW in Oman, with more than 60% taking iron and folate supplements, while the proportions amongst WRA were considerably lower (less than 10% for folic acid, vitamin A, vitamin D, and multivitamins, and approximately 11% for iron) [36]. In Morocco, however, the coverage of WRA with iron and folic acid supplementation was reported at 73.5% [84]. In Egypt, according to the 2014 Demographic and Health Survey, 72% of mothers who had four or more antenatal care visits prior to their last birth were given or had bought iron supplements during their pregnancy, compared with 54% of mothers who had one to three visits, and only 26% of those who had no antenatal care [85]. Using self-reported data, Ibrahim et al. showed that 51.1% of PW in Egypt did not adhere to folic acid and iron supplementation, while a pill count assessment revealed even higher levels of non-adherence (63.3%) [86]. Studies conducted in countries of the Gulf and Levant regions indicated that 6–45% of PW reported taking folic acid supplementation during their first-trimester, with the lowest rate being reported from Lebanon (6–14% of women) [87,88,89,90]. In the 2013 Palestinian Micronutrient Survey, the regular intake of iron and folate supplements amongst PW was 64.9% in the Gaza Strip and 72.9% in the West Bank [65].

Some countries have also developed and implemented supplementation programs that target other age groups. For instance, in 2020, Pakistan launched the Adolescent Nutrition Supplementation Guidelines for the country, which recommend screening for anemia in adolescents, providing them with daily iron and folic acid supplementation, as well as nutritional counseling [91]. In Afghanistan, weekly iron folic acid supplementation of adolescent girls (10–19 years old) was launched in March 2016, with 1.6 million adolescent girls under coverage of this program [92]. Palestine is implementing a supplementation program with vitamin A for infants up to one year of age. The 2013 Palestinian Micronutrient Survey showed that 84.4% of participating children had in fact received vitamin A and D supplements (72% in Gaza Strip vs. 97.3% in West Bank) [65]. Vitamin A supplementation programs of children under two years of age are also being implemented in Oman and Morocco (82% coverage) [84,93]. In Jordan, children aged 18 months attending clinics for measles, mumps, and rubella (MMR) vaccination have been routinely given a vitamin A capsule (200,000 IU) since 2010 [94]. Egypt maintains a vitamin A supplementation program for U5C, starting at nine months of age. However, compliance is poor, where only 44% and 49% of children aged 9–11 and 18–27 months were reported to receive this supplement, respectively [95]. Oman and Somalia are also implementing vitamin A supplementation programs targeting postpartum women [93,96].

#### 5.3.3. Nutrition Education and Awareness

The literature suggests that proper education can achieve better rates of anemia reduction than the provision of supplements alone [2,97]. In the EMR, comprehensive education programs focusing on anemia per se are nonexistent. However, several countries have developed food-based dietary guidelines (FBDGs) that can be used in nutrition education and media campaigns to enhance the population’s nutritional knowledge. More specifically, eight countries in the region have developed FBDGs, including Afghanistan, Iran, Lebanon, Morocco, Oman, Qatar, Saudi Arabia [98,99,100,101,102,103,104,105], and, most recently, Jordan [106]. Seven FBDGs (Afghanistan, Iran, Jordan, Lebanon, Morocco, Oman, and Qatar) provide information related to micronutrient intakes for normal metabolic growth and physical well-being. Afghanistan, Jordan, and Lebanon recommend consuming fortified foods, such as vitamin D–fortified foods (e.g., milk and yogurt), iron-fortified flour, and vitamin A–fortified oil. The FBDGs for Saudi Arabia and Afghanistan include recommendations to prevent micronutrient deficiencies (such as iron, vitamin A) in children [98,99,101]. Advice on ways to enhance iron and folic acid intakes is included in the majority of these guidelines as a strategy to prevent anemia. It is important to mention that there is no data on the dissemination of these FBDGs within countries or whether these guidelines have had any impact on raising nutrition awareness in the population.

### 5.4. Interventions to Address Non-Nutritional Causes of Anemia in the EMR

The non-nutritional causes or contributing factors to anemia comprise acute and chronic parasitic infestations (e.g., malaria and infestation by soil-transmitted Helminth Schistosomiasis) and genetic disorders, such as thalassemia, glucose-6-phosphate dehydrogenase (G6PD) deficiency, and sickle cell trait. Environmental contributors include inadequate sanitation, poor personal hygiene, and lack of access to clean and safe water, in addition to deficiencies in economic, political, and institutional capacities and/or resources [2,19,20,22,118]. Other factors linked to anemia, especially in women, encompass poverty, low literacy, social norms, lack of empowerment, poor nutrition knowledge, inadequate access to healthcare, and poor maternal and child care, as well as living in rural areas [2,19,20].

#### 5.4.1. Malaria Control Programs

In 2015, it was estimated that 44% of the EMR population live in areas at risk of local transmission of malaria [119]. The hemolysis induced by malarial parasites is one of the main mechanisms through which malaria causes anemia [2,120]. In addition, the imbalance between the pro- and anti-inflammatory pathways causes a modification in erythroid cell proliferation, which may lead to severe malarial anemia [121]. The EMR comprises three different ecoepidemiological zones, and encompasses countries with significant disparities in socioeconomic development [119,122,123], a diversity that undoubtedly impacts the effectiveness of malaria control in different countries [122]. To reflect these variations, countries are classified into three main groups. Group 1 includes countries with a high burden of malaria, comprising Afghanistan, Djibouti, Pakistan, Somalia, Sudan, and Yemen [119]. Countries belonging to this group constitute approximately 44% of the region’s population, and cover areas that have a high risk of malaria transmission or are menaced by epidemics and/or complex emergency situations [122]. Between 2000 and 2010, Afghanistan has achieved a more than 50% reduction in confirmed malaria cases [124]. In contrast, Djibouti, Pakistan, Somalia, Sudan, and Yemen have not witnessed a consistent reduction in the number of malaria cases, although regional variations within countries have been reported [124]. Inadequacy in human resources and capacity is a main limitation hindering progress and challenging the implementation and sustainability of malaria control programs in Group 1 countries [125]. Group 2 countries are classified as those that have low malaria transmission and are targeting its elimination. This group of countries includes Iran and Saudi Arabia, which represent 17% of the region’s population [119]. Group 2 countries were able to achieve a continuous and consistent decrease in malaria incidence over the past ten years. The malaria control programs implemented in these countries were shown to be sustainable and to benefit from significant political and financial support at the national level [122]. Group 3 includes countries that have eliminated malaria decades ago, such as Bahrain, Jordan, Kuwait, Lebanon, Libya, Palestine, Qatar, and Tunisia, and countries that have accomplished certification of malaria elimination during the past ten years, such as Morocco (2010) and the UAE (2007) [119]. This group also comprises countries where local transmission has been interrupted for at least three years within the past decade, but where elimination has not yet been certified, including Egypt, Iraq, Oman, and the Syrian Arab Republic [126]. In all Group 3 countries, a limited number of local cases may still occur because of importation; but the well-founded malaria programs are able to respond and prevent the re-establishment of local transmission [122].

At the regional level, the goal that was set by the Regional Malaria Action is: “By 2030, to interrupt malaria transmission in areas where it is feasible and reduce the burden by more than 90% in areas where elimination is not immediately possible, so that malaria is no longer a public health problem or a barrier to social and economic development” [119]. Specific objectives include reducing, by the end of 2020, the incidence of malaria by more than 40% compared to 2015. These strategic regional goals and targets, if reached, will contribute towards additional reductions in the prevalence of anemia in the region.

#### 5.4.2. Soil-Transmitted Helminth Infections

Soil-transmitted helminthiasis (STH) is included amongst the five neglected tropical diseases in the EMR region, and there is a focus on its eradication or elimination as a public health problem [127]. STHs are transmitted via eggs that are passed in human feces, eventually getting deposited on soil and leading to contamination in areas with poor sanitation [128]. The most common infections are caused by hookworms, roundworms, and whipworms [2,128,129]. Hookworm infection is a main cause of anemia, given the blood loss that accompanies the damage induced by hookworms’ feeding on the gastro-intestinal mucosa. Roundworms lead to intestinal inflammation, coupled with poor nutrient absorption, and consequently nutritional deficiencies. Whipworms adversely affect fat digestion, and lead to vitamin malabsorption, poor appetite, blood loss, and associated anemia [2,128,129]. The WHO recommends periodic medicinal treatment (deworming) to all at-risk population groups living in endemic areas [128].

Preventive chemotherapy programs conducted in the region are mainly focusing on school-aged children to control STH. School-based programs have been implemented in Afghanistan, Egypt, Iraq, Pakistan, Somalia, Sudan, the Syrian Arab Republic, and Yemen, as well as amongst refugee populations under the United Nations Relief and Works Agency for Palestine Refugees in the Near East (UNRWA), reaching 10.8 million school-aged children in 2018 [127]. The total number of school-aged children requiring preventive chemotherapy in 2018 was estimated at 43.3 million in the region. Afghanistan and the Syrian Arab Republic implemented preventive chemotherapy for school-aged children, reaching more than 75% national coverage in 2018 [127,130]. In contrast, other endemic countries in the region, including Djibouti, Pakistan, Sudan, and Yemen, did not report effective national coverage amongst school-aged children [131]. The monitoring framework for neglected tropical diseases in the EMR has set a target to increase the number of countries attaining at least 75% deworming coverage of school-aged children from two in 2018 to four in 2021 [30].

In addition to school-based programs, Afghanistan has implemented a preventive program targeting preschool children, with national coverage increasing over time [131]. However, in 2018, the treatment campaign was not implemented because of challenges related to the procurement of anthelmintic and the nonexistence of donation programs for this age group [131]. In addition, the NNS conducted in 2018 in Pakistan showed that only 13.1% of 24–59 month old children had been given deworming medications in the six-month period preceding the survey [32]. Data amongst WRA is relatively scarce. It was reported that only 3.8% of women in Afghanistan had taken deworming medicines during their last pregnancy [33]. There is an urgent need to upscale preventive programs to include preschool children and WRA [131], and to align national strategies, particularly in endemic countries, with the WHO targets for STH prevention and control by 2030, which include: “Achieve and maintain elimination of STH morbidity in preschool- and school-age children” and “Establish efficient STH control programmes in adolescent, pregnant and lactating women of reproductive age.”

#### 5.4.3. WASH in Countries of the EMR

Poor access to safe drinking water and inadequate sanitation and hygiene can have severe adverse consequences on child nutritional status and anemia risk through their effect on diarrheal diseases, gastro-intestinal parasites, and environmental enteropathy [24]. The region harbors countries with significant differences in WASH. While 87% and 73% of the region’s population have access to basic drinking water and basic sanitation, respectively, the safe delivery of these services is not always managed as per the SDG targets. In addition, approximately 13% (84.4 million people) of the region’s population continue to be without basic water services, with the vast majority (64.6 million) living in Afghanistan, Pakistan, Sudan, Somalia, and Yemen, and 15 million living in Iran, Iraq, and Morocco. Around 27% of the population (175 million people) continue to be without basic sanitation services. Of these, 154 million reside in Afghanistan, Iran, Pakistan, Somalia, Sudan, and Yemen, and 17 million live in Egypt, Iraq, and Morocco [132].

Good hygiene is considered amongst the simplest and most efficient means to protect against the spread of infectious diseases and provide a supportive environment for nutrition [132,133]. The SDGs specified, for the first time, an indicator pertinent to the proportions of people with home access to hand washing facilities. Information on the availability of hygiene facilities is limited in the EMR, with data on handwashing with soap and water being available for only eight of the 22 countries (Afghanistan, Egypt, Iraq, Pakistan, Somalia, Sudan, Tunisia, and Yemen). Accordingly, access to water and soap for handwashing fluctuated considerably between countries, varying from 10% in Somalia to approximately 90% in Egypt, Iraq, and Tunisia [132]. It is important to note that variations may exist within the same country, particularly when comparing urban and rural areas. In general, the use of untreated surface water from irrigation channels, lakes, or rivers is much more common in rural areas compared to urban ones [132,134].

#### 5.4.4. Genetic Disorders Leading to Anemia

Consanguinity represents the chief risk factor for genetic disorders in the region, and there is a lack of proper counseling services for couples [135]. Depending on the area and the country studied, between 20% and 70% of families in the EMR are consanguineous [136,137], and first-cousin marriages are very common traditionally [136,138]. This consanguinity contributes towards increasing the frequency of homozygosity for autosomal recessive conditions [136,137]. The autosomal recessive disorders that are common in countries of the region include alpha thalassemia (carrier rate varies between 2–50%), beta thalassemia (carrier rate varies between 2–7%), and sickle cell anemia (carrier rate varies between 0.3–30%) [135,139,140]. Deficiency of G6PD (estimated at 24% amongst male Bahrainis and 6–13% amongst male Saudis) [136] may also contribute to anemia in the region, owing to its impact on oxidative stress and hemolysis [136,141]. The expert meeting report on the prevention of congenital and genetic disorders in the EMR [135] has called for interventions during the preconception period, targeting all women of childbearing age, including the screening for carriers of common autosomal recessive disorders in the region, such as beta thalassemia and sickle cell anemia [135].

#### 5.4.5. Environmental and Socioeconomic Domains

According to the WHO, it is crucial to consider environmental and socioeconomic factors within the determinants of anemia, and hence within the comprehensive package of anemia reduction interventions. Factors that may impact anemia prevalence and affect the quality and success of anemia reduction efforts in the EMR may include poverty alleviation, social protection, women’s empowerment, quality of healthcare, and coverage, as well as distressed health and environmental contexts [2,142]. The pathways between these factors and anemia status are complex, and often involve context-specific sociopolitical processes and household-level environmental factors that may affect health and disease outcomes [143,144,145,146,147,148]. Although it is well-recognized that such socioeconomic and environmental factors are critical to health in general, data on their direct link to anemia is often limited at the national, regional, or even global level [2].

Acknowledging that the EMR harbors countries with significant disparities in socioeconomic development, as well countries with considerable distress resulting from political turmoil or conflict, it is important that national anemia reduction strategies tailor for the local socioeconomic and environmental context in their anemia reduction efforts. These may include the design and implementation of social policies and social protection schemes targeting low income, resource poor, food insecure, economically marginalized, and socially excluded population groups (especially women and children) [149]. For instance, cash transfer programming, which is one of the most widely implemented social protection schemes in the context of conflict and displacement, was shown to improve a household’s access to food and healthcare and potentially contribute to improved health nutrition and decreased risk of anemia in women and young children [150]. Other interventions may include the development of context-relevant women’s empowerment interventions, which have the potential to improve not only women’s health (and anemia) status, but also the nutritional status of their children [2,151], engaging women and their communities in anemia reduction programming to improve impact, and ensuring universal healthcare for better health outcomes [2,152].

### 5.5. Governance: Anemia Reduction Programs and Coordination

The review of evidence undertaken in this paper showed that none of the EMR countries have a comprehensive anemia reduction strategy. Instead, anemia reduction initiatives and activities are fragmented, with no strategic outlook or planning. In many of the countries, anemia is often addressed in national nutrition strategies and action plans with a focus on IDA, hence missing on the opportunity to address other potential causes of anemia in the local context. The multifactorial nature of anemia and the need for interventions that tackle the broad scope of its potential determinants highlight the need for multisector initiatives and resources to bolster and safeguard optimal effectiveness of national anemia reduction activities [2,17]. This emphasizes the significant importance of cross-sectoral collaboration, support, and capacity-building to ensure the sustainability and success of anemia reduction efforts [2].

## 6. Discussion and the Way Forward

This review showed that the majority of countries of the EMR are not on course to meet the target of reducing anemia by 50% in WRA by 2025 [11]. Amongst those countries in which some progress has been made, Oman, Pakistan, and the Syrian Arab Republic were found to be on track, while Egypt was considered in the right direction. In addition, the majority of countries did not achieve any reduction in the prevalence of anemia amongst other vulnerable population groups, such as U5C. This paper showed that IDA contributed to less than half of the regional burden of anemia, while shedding light on other potential nutritional and non-nutritional contributors to anemia in the region. By reviewing the interventions currently implemented in countries of the region, this paper provided evidence on the lack of comprehensive anemia reduction strategies in countries of the region, with interventions often tackling one determinant at a time, instead of holistically addressing the multifactorial etiology of anemia.

Despite the common misconception that iron deficiency is equivalent to anemia, this review showed that IDA contributed to less than half of the regional burden of anemia. These findings may be limited by the scarcity of data on IDA, particularly amongst PW. In addition, inflammation, which may affect the levels of SF, was not assessed in some of the studies reporting on IDA, which could have led to errors in its identification [35,46,47,52]. Despite these limitations, the findings that IDA contributed to less than half of anemia in the EMR are in agreement with those reported by a meta-analysis of nationally representative surveys from 23 countries, showing that the proportion of anemia associated with iron deficiency in WRA was estimated at 37% on average [153]. Indeed, it is important to reiterate that the causes of anemia are complex and multifactorial, and go far beyond the deficiency of iron [2,17,19,20]. Additional determinants of anemia in the region may include micronutrient deficiencies, such as vitamins A, B12, folate, and zinc, which were found to be prevalent in the population, although the available data is scarce. Non-nutritional causes, such as parasites, infections, poor sanitation, and genetic disorders/hemoglobinopathies, coupled with distressed environmental and social factors, may also be contributing to the high burden of anemia in the EMR.

Countries of the region have developed and implemented various interventions to tackle the persistent burden of anemia. The implemented nutrition-specific interventions include food fortification, supplementation, and nutrition education programs. Despite the fact that the majority of countries have ongoing fortification programs, very few were able to achieve progress in anemia reduction. This poor progress may be a reflection of the numerous limitations/challenges related to the implementation and monitoring of fortification programs in the region. Although some countries have adopted the recommended NaFeEDTA as the main wheat flour fortifier (e.g., Afghanistan, Morocco), the majority are still using poorly bioavailable ferrous sulfate or electrolyte iron compounds [54]. Other limitations of the ongoing fortification programs include the use of inadequate levels of iron compounds, as well as poor legislation and monitoring systems [54,154,155]. As for supplementation programs, these are also common in the region, with the majority of countries targeting PW [83], while less than half have also targeted WRA [83], U5C, or adolescent girls [65,84,91,92,93,94,95]. Of interest is the fact that, even amongst countries that have implemented supplementation programs, poor or no progress was observed in relation to anemia prevalence. It is important to mention that the success of these programs may be limited by challenges related to the distribution and cost of supplements, which hamper the programs’ uptake and effectiveness [79]. In fact, several studies conducted in the region have highlighted poor uptake of these supplementation programs and low supplement use amongst PW and WRA [33,85,86,87,88,89,90,95]. In addition, although nutrition education programs, such as FBDGs, are common in the EMR, there is a lack of nutrition education programs that are particularly focused on anemia prevention and alleviation.

Interventions to address the non-nutritional causes of anemia have also been reviewed in this paper, with significant disparities noted between countries. For instance, the implementation of malaria control programs had varying degrees of effectiveness and success between Member States. While Group 1 countries remain threatened by local malaria transmission, Group 2 and Group 3 countries have either experienced low numbers of malaria cases or successfully eliminated it decades ago [119,122]. As for the burden of STH infections, although people at risk encompass preschool children, school-aged children, WRA, PW, and breastfeeding women, preventive chemotherapy programs conducted in the region remain focused on school-aged children. Significant disparities in the coverage of these preventive programs were also noticed between countries [127,130,131]. The main challenges in the implementation of these programs seem to be related to procurement issues, as well as the lack of sustainable donation programs [131]. Interventions focusing on the improvement of sanitation and good hygiene to limit the spread of infectious diseases, and hence anemia, have been implemented in various countries of the region. Although data on these indicators remain scarce, current estimates show that access to water and soap for handwashing fluctuated considerably between countries, varying from 10% in Somalia to approximately 90% in Egypt, Iraq, and Tunisia [132]. In this context, the Strategy on Nutrition for the EMR 2020–2030 included the improvement of water, sanitation, and hygiene as one of its priority actions for securing safe and supportive environments for nutrition at all ages [24]. Another potential factor that may be contributing to anemia in the region is the burden of genetic disorders. Despite consanguinous marriages being common in various countries of the EMR [136], there is little being done on raising awareness or providing proper counseling for couples. The expert meeting report on the prevention of congenital and genetic disorders in the EMR has called for more studies and country-specific data to better understand the burden of genetic disorders in individual countries, while also calling for national policy development to make sure that genetic disorders are prioritized and incorporated within the basic health service packages offered by primary healthcare centers [135].

**Figure 5:**
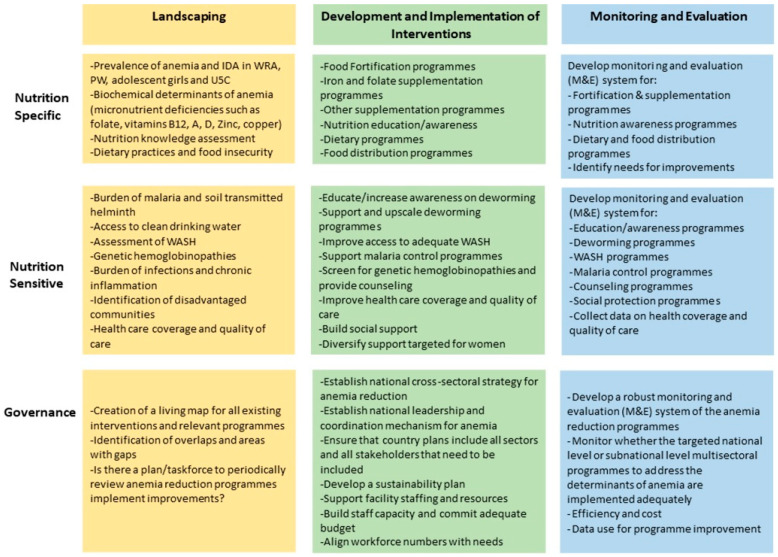
A framework for action for optimizing anemia reduction efforts in countries of the EMR. Abbreviations: EMR: Eastern Mediterranean Region; IDA: iron deficiency anemia; PW: pregnant women; U5C: underfive children; WASH: Water, Sanitation, and Hygiene; WRA: women of reproductive age.

The review of evidence undertaken in this paper showed that anemia reduction interventions are fragmented, and none of the EMR countries have a comprehensive anemia reduction strategy. Acknowledging that the control of anemia is crucial for the prevention of ill health amongst women, children, and future generations, the Strategy on Nutrition for the EMR 2020–2030 has called for action to reduce the burden of anemia in countries of the region [24]. In this context, it is crucial that countries of the EMR revisit their national anemia reduction activities by adopting holistic and comprehensive approaches that consider all domains and procedures that may have a role in alleviating anemia [2]. Policymakers seeking to reduce the prevalence of anemia in countries of the region will not achieve the desired outcomes without first understanding the local context and intervening through a broad range of pertinent interventions that address this complexity [2,142]. This can be done by undertaking local landscape analyses to identify the key drivers of anemia, understanding which anemia-related programs are working in the country and which are likely bottlenecks, and delineate opportunities for multisectoral support and engagement. Landscape analyses can be used to inform decisions and develop context-specific interventions (Figure 5) [2,156].

The package of interventions to be developed should include nutrition-specific as well as nutrition-sensitive interventions, while allowing for intersectoral coordination and clear governance. Recommendations for countries of the region may therefore be guided by the framework of action presented in Figure 5 to develop national anemia reduction strategies that include a portfolio of policies and interventions, such as the improvement of identification and measurement of anemia in vulnerable population groups; scaling up the coverage of prevention and treatment activities; revision of food fortification programs at country levels, including the use of the recommended NaFeEDTA iron compound in wheat flour at adequate levels; improved legislation and monitoring of fortification programs; adoption of intermittent iron and folic acid supplementation in WRA to reduce the risk of anemia in populations where the prevalence of anemia amongst WRA is 20% or higher [157]; adoption of multiple micronutrient supplements in countries where micronutrient deficiencies are prevalent; ensuring a more efficient distribution of supplements at reasonable costs; community mobilization and social marketing to raise awareness pertinent to iron supplementation in WRA; implementation of adequate measures to enhance WASH and prevent parasitic infections; the design and implementation of social policies and social protection schemes; and creation of partnerships between state and non-state actors to ensure financial commitment towards anemia reduction strategies.

The combination of interventions and activities must be evaluated according to the best practices of monitoring and evaluation, with intermediate impact assessment as well as outcome evaluation (Figure 5) [2,156]. The monitoring of interventions would necessitate the collection of data on the intervention quality and completeness, coverage, and demand of the intervention, as well as efficiency and cost, while building on the collected data for program improvement. The type of evaluation to implement ought to be determined, an evaluation plan should be put in place (including indicators, data sources, methods, and a timeline), and all resources needed to implement the evaluation should be identified.

In the development, implementation, and monitoring of anemia reduction interventions, countries of the EMR should strive to establish national leadership and coordination mechanisms for anemia reduction. Guidelines for governmental efforts in anemia reduction should be built around several action areas, mainly the creation of a supportive environment for the execution of comprehensive food policies for nutrition and nutrition-sensitive actions that facilitate the prevention and control of anemia amongst all age groups, including WRA and U5C; the inclusion of all of the needed effective health interventions with a potential influence on anemia in country-based strategic plans; the provision of all required human and financial resources for the execution of anemia reduction activities; the monitoring and evaluation of the implementation of all related policies and programs [2,158]; harmonization of reporting between stakeholders; and commitment to a coordinating body that receives support from the highest level of ministries and governments [2,159,160]. The adoption of intersectional approaches may in fact require changes in the way the health sector has worked in the past to comprise open multi-stakeholder engagement and support [2,161].

## 7. Conclusions

Although this review may have been limited by the scarcity of data, it has allowed the assessment of the burden of anemia and its temporal trend amongst vulnerable population groups in the EMR. It showed that, except for Oman, Pakistan, the Syrian Arab Republic, and Egypt, where some progress was made, the majority of countries in the region are not on track towards meeting the WHA and SDG targets. It has also documented that iron deficiency is not the sole factor leading to anemia in countries of the region, highlighting the need for a more holistic approach in tackling the persistent burden of anemia in countries of the region. The review has also identified many gaps and/or problems with anemia reduction efforts in the region, described various components required for successful evidence-informed anemia reduction programs, and highlighted that the optimal portfolio of anemia reduction interventions should include both nutrition-sensitive and nutrition-specific interventions, while ensuring multisectoral integrated support. It is through such a multi-perspective and coordinated approach that countries of the region will be able to reduce anemia and thus meet the targets set by the WHA [11] and the SDGs [16].

## Figures and Tables

**Table 1:** Prevalence of anemia amongst women and U5C in the EMR (based on latest available estimates).

Country	Prevalence of Anemia in PW (%) ^a^	Prevalence of Anemia in WRA (%)	Prevalence of Anemia in U5C (%) ^c^
Afghanistan	38.2	42 ^a^	46.4
Bahrain	42.8	45.9 ^b^	30.1
Djibouti	37.6	32.7 ^a^	42
Egypt	22.6	28.5 ^a^	31.7
Iran	34.1	30.5 ^a^	28.2
Iraq	33.5	29.1 ^a^	24.1
Jordan	37.1	34.7 ^a^	31.1
Kuwait	31.2	37.8 ^b^	25.3
Lebanon	35.2	31.2 ^a^	25
Libya	38	32.5 ^a^	28.8
Morocco *	40.4	34.4 *	47.5 *
Oman *	29.3 *	27.8 *	23.8 *
Pakistan *	51.3	42.7 *	53.7 *
Palestine *	30.5 *	27 ^b^	--
Qatar	33.4	27.7 ^a^	26.3
Saudi Arabia	45.5	43.7 ^b^	37.8
Somalia	46.8	44.4 ^a^	55.8
Sudan *	34.1	30.7 ^a^	48.1 *
Syrian Arab Republic	36.1	33.6 ^a^	34.9
Tunisia	36.7	31.2 ^a^	28.8
UAE	33.2	27.8 ^a^	27.2
Yemen	63	69.6 ^a^	83.5

^a^ Data from the WHO GHO Data Repository–Prevalence of anemia in women (2016) [27]. ^b^ Data from the WHO EMRO-Monitoring health and health system performance in the Eastern Mediterranean Region Core indicators and indicators on the health-related Sustainable Development Goals (2019) [30]. ^c^ Data from the WHO GHO Data Repository–Prevalence of anemia in U5C (2016) [28]. * The data for WRA and U5C in Morocco were based on the recent Moroccan NNS 2019–2020 [37]. The data for Oman were based on the recent Oman NNS in 2017 [36]. The data pertinent to WRA and U5C in Pakistan were based on the recent NNS 2018 [32]. The data pertinent to PW in Palestine was based on the recent Palestinian National Nutrition Surveillance Survey 2018 [38]. The data for U5C in Sudan were based on the recent Simple Spatial Survey Method (S3M II) 2020 [39]. Abbreviations: EMR: Eastern Mediterranean Region; EMRO: Regional Office for the Eastern Mediterranean; GHO: Global Health Observatory; NNS: National Nutrition Survey; PW: pregnant women; U5C: underfive children; UAE: United Arab Emirates; WHO: World Health Organization; WRA: women of reproductive age.

**Table 2:** Comparison of the public health significance of the burden of anemia vs. IDA based on studies that have reported on both.

Indicator	Normal (≤4.9%)	Mild (5.0–19.9%)	Moderate (20.0–39.9%)	Severe (≥40.0%)
Underfive Children				
Anemia		Jordan [42]	Oman [36] Qatar [47] UAE (Al Ain) [51]	Afghanistan [33] Iran (South West) [49] Morocco [37] Pakistan [32]
IDA	Jordan [42] Oman [36]	Afghanistan [33] Morocco [37] Qatar [47] UAE (Al Ain) [51]	Iran (South West) [49] Pakistan [32]	
Women of Reproductive Age				
Anemia			Jordan [42] Oman [36] Morocco [37]	Afghanistan [33] Pakistan [32]
IDA		Afghanistan [33] Jordan [42] Morocco [37] Oman [36] Pakistan [32]		

The classification of the public health significance for Afghanistan, Iran, Jordan, Qatar, and the UAE was based on the values reported in Figure 1 (i.e., based on studies that have reported on both IDA and anemia). Hence, the values and their significance may differ from those included in Table 1. Abbreviations: IDA: iron deficiency anemia; UAE: United Arab Emirates.

**Table 3:** Nutrition-specific interventions to reduce anemia in countries of the EMR.

Country	Iron/FA Supplementation in PW ^a^	Supplementation in U5C	Wheat Flour Fortification ^b^	Other Food Fortifications
Afghanistan	Yes	Iron for children under 24 months [107]. Multiple micronutrient for infants 6–23 months (powders containing iron, FA, zinc, and vitamins A, B, and D [108]). Vitamin A in children 6–59 months [107]. Zinc in preschoolers with diarrhea [107].	Mandatory—2018 (Iron, FA, and vitamins B12 and A) [54].	Cooking oil and ghee –Mandatory–2018 (Vitamins A and D) [107].
Bahrain	Yes	ND	Mandatory–2002 (Iron and FA) [54].	ND
Djibouti	ND	ND	Mandatory–2013 (Iron, FA, and zinc) [54,109].	ND
Egypt	Yes	ND	Voluntary–2009 (Iron and FA) [54,110].	Vegetable oils–2009 [111].
Iran	Yes	Iron for children 6 to 24 months [112]. Multivitamin/vitamin A + D drop from day three of birth to 24 months [112].	Mandatory–2007 (Iron, FA-proposed for vitamin D and zinc) [54].	ND
Iraq	Yes	ND	Mandatory–2008; Government provision of premix stalled since 2014 (Iron and FA) [54].	ND
Jordan	Yes	Vitamin A capsule for children at the age of 18 months (200,000 IU) [94].	Mandatory–2002 (Iron, FA, zinc, and vitamins A, D, B1, B2, B3, B6, and B12) [42,54].	ND
Kuwait	Yes	ND	Mandatory (Iron, FA, and vitamins B1, B2, and B3) [54].	ND
Lebanon	Yes	ND	No fortification	ND
Libya	Yes	ND	No fortification	ND
Morocco	Yes	2014–Vitamin A for children under the age of 2 [84].	Mandatory–2006 (Iron and FA) [54,113].	Vegetable oils–2006 [114].
Oman	Yes	1998–Vitamin A for children under the age of 2 [93].	Mandatory–1996 (Iron and FA) [54].	Vegetable oils–Mandatory–2010 (Vitamins A and D) [36].
Pakistan	Yes	ND	Mandatory in some provinces–2017 (Iron, FA, zinc, and vitamin B12) [54].	Oils and ghee–2016 (Vitamins A and D) [115].
Palestine	Yes	Iron drops for infants up to 24 months [65]. Vitamin A and D for infants from birth and until 12 months [65].	Mandatory–2006 (Iron, FA, zinc, and vitamins A, D, B1, B2, B3, B6, and B12) [54,65].	ND
Qatar	Yes	ND	Voluntary (Iron, FA, and vitamins B1, B2, and B3) [54].	ND
Saudi Arabia	Yes	ND	Mandatory (Iron, FA, and vitamins B1, B2, and B3 [54].	ND
Somalia	Yes Supplements also include vitamins A, B1, B2, B6, B12, C, D, and E, niacin, copper, selenium, iodine, and zinc.	Iron syrups for anemia treatment in young children [96]. Vitamin A for U5C [96]. Zinc for U5C for the treatment of diarrhea [96].	ND	ND
Sudan	Yes	Vitamin A for 6–59 months during the 2019 measle campaign [116]. Multiple Micronutrient Powder (called Vitamino) (15 micronutrients including Iron and FA) for 6–59 months [116].	Voluntary by one mill–2005 (Iron and FA) [54].	ND
Syrian Arab Republic	Yes	ND	Voluntary (Iron and FA) [54].	ND
Tunisia	Yes	ND	ND	ND
UAE	Yes	ND	Voluntary (Iron, FA, and vitamins B1, B2, and B3) [54].	ND
Yemen	ND	ND	Mandatory–2001 (Iron and FA) [54].	Oils and ghee–2001 (Vitamins A and D) [117].

^a^ Data on iron/folate supplementation in PW were obtained from the Global Nutrition Policy Review 2016–2017 dataset [83]. ^b^ Data on wheat flour fortification were obtained from the Wheat Flour Fortification Report by Al Jawaldeh et al. 2019 [54], as well as country-specific references as indicated in the table. Abbreviations: EMR: Eastern Mediterranean Region; FA: folic acid; ND: No data; PW: pregnant women; U5C: underfive children; UAE: United Arab Emirates.

## Data Availability

The datasets used and/or analyzed during the current study are available from the corresponding author on reasonable request.

## Abbreviations

EMR: Eastern Mediterranean Region; EMRO: Regional Office for the Eastern Mediterranean; FBDGs: food-based dietary guidelines; G6PD: glucose-6-phosphate dehydrogenase; Global Health Observatory; GINA: Global Database on the Implementation of Nutrition Action; Hb: hemoglobin; IDA: iron deficiency anemia; MMR: measles, mumps, rubella; NNS: National Nutrition Survey; PW: pregnant women; SDGs: Sustainable Development Goals; SF: serum ferritin; STH: soil-transmitted helminthiasis; U5C: under five children; UAE: United Arab Emirates; UNRWA: United Nations Relief and Works Agency; VAD: vitamin A deficiency; VDD: vitamin D deficiency; WASH: water, sanitation and hygiene; WHA: World Health Assembly; WHO: World Health Organization; WRA: women of reproductive age.
